# Ethanol Induces Neuroinflammation in a Chronic Plus Binge Mouse Model of Alcohol Use Disorder via TLR4 and MyD88-Dependent Signaling

**DOI:** 10.3390/cells12162109

**Published:** 2023-08-21

**Authors:** Kalee N. Holloway, James C. Douglas, Tonya M. Rafferty, Cynthia J. M. Kane, Paul D. Drew

**Affiliations:** 1 Department of Neurobiology and Developmental Sciences, University of Arkansas for Medical Sciences, Little Rock, AR 72205, USA; 2 Department of Neurology, University of Arkansas for Medical Sciences, Little Rock, AR 72205, USA

**Keywords:** AUD, neuroinflammation, TLR4, MyD88, TRIF, ethanol

## Abstract

Ethanol induces neuroinflammation, which is believed to contribute to the pathogenesis of alcohol use disorder (AUD). Toll-like receptors (TLRs) are a group of pattern recognition receptors (PRRs) expressed on both immune cells, including microglia and astrocytes, and non-immune cells in the central nervous system (CNS). Studies have shown that alcohol activates TLR4 signaling, resulting in the induction of pro-inflammatory cytokines and chemokines in the CNS. However, the effect of alcohol on signaling pathways downstream of TLR4, such as MyD88 and TRIF (TICAM) signaling, has not been evaluated extensively. In the current study, we treated male wild-type, TLR4-, MyD88-, and TRIF-deficient mice using a chronic plus binge mouse model of AUD. Evaluation of mRNA expression by qRT-PCR revealed that ethanol increased IL-1β, TNF-α, CCL2, COX2, FosB, and JunB in the cerebellum in wild-type and TRIF-deficient mice, while ethanol generally did not increase the expression of these molecules in TLR4- and MyD88-deficient mice. Furthermore, IRF3, IRF7, and IFN-β1, which are associated with the TRIF-dependent signaling cascade, were largely unaffected by alcohol. Collectively, these results suggest that the TLR4 and downstream MyD88-dependent signaling pathways are essential in ethanol-induced neuroinflammation in this mouse model of AUD.

## 1. Introduction

Alcohol use disorder (AUD), a neurological disorder common in adults, has a remarkable impact at the individual and societal level. The annual cost of excessive alcohol use in the United States has been estimated to be approximately $249 billion [1]. In 2019, 14.1 million adults ages 18 and older were diagnosed with AUD [2]. AUD can range from mild to moderate to severe; it can affect a variety of organ systems, including the central nervous system (CNS); and the consequences of AUD can last a lifetime. Although there are a variety of treatment strategies for AUD, such as medications, behavioral therapy, and mutual-support groups, the efficacy of these therapies is limited, and individuals with AUD are susceptible to relapse.

Animal models of AUD have proven extremely valuable, as they exhibit similar neuropathology and behavioral deficits observed in humans with AUD [3]. In these animal models, ethanol exposure during adulthood elicits neuroinflammation, which is believed to promote ethanol-induced neuropathology and associated behavioral aberrations such as cognitive dysfunction and increased drinking behavior [4,5,6,7,8,9,10,11,12,13].

**Figure 1:**
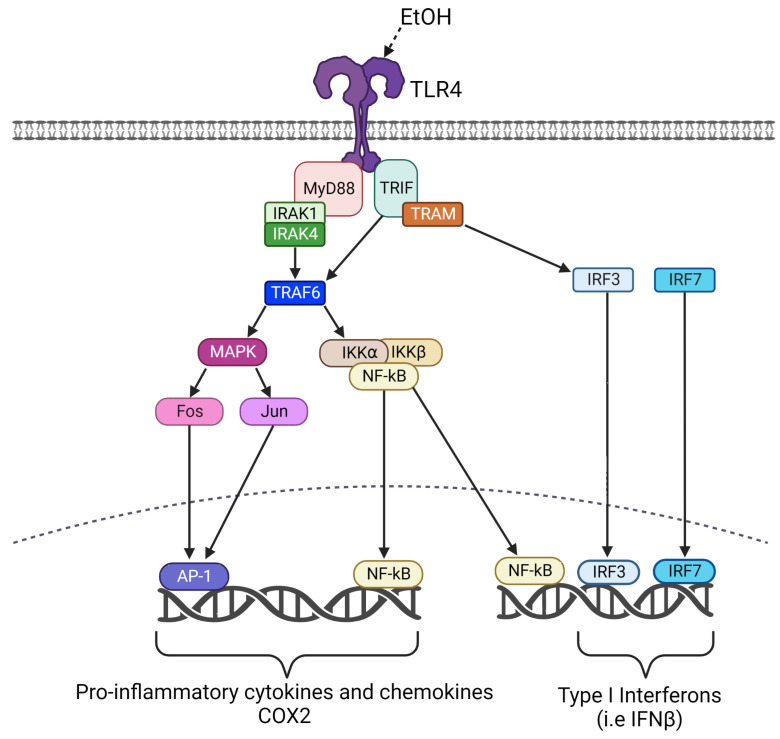
Schematic representation of the TLR4 signaling pathway.

Microglia and astrocytes are resident glial cells that play critical roles in neuroinflammation and can protect the CNS from insults, including pathogens. However, chronic neuroinflammation is believed to contribute to neuropathology in a variety of disorders, including AUD [14,15,16,17]. Toll-like receptors (TLRs) are a family of evolutionarily conserved proteins that play critical roles in immune responses as well as responses to cell damage [18]. They are type I transmembrane proteins whose extracellular domains bind to pattern-associated molecular patterns (PAMPs) found on pathogens, and damage-associated molecular pathogens (DAMPs) produced by cell damage. TLRs initiate signal transduction pathways through intracellular toll/IL-1 receptor-like (TIR) domains [19,20]. Previous studies have suggested that TLR4 is a critical modulator of ethanol-induced neuroinflammation. For example, ethanol resulted in the release of endogenous danger signals in the CNS, which triggered TLR4 signaling [21]. Ethanol also induced the expression of a variety of pro-inflammatory molecules, including tumor necrosis factor alpha (TNF-α), interleukin 1 beta (IL-1β), NF-ĸB, inducible nitric oxide synthase (iNOS), and cyclooxygenase-2 (COX2) in wild-type but not TLR4-deficient mice [5,22]. Previous studies have begun to evaluate the effects of ethanol on signaling pathways downstream of TLR4, such as MyD88 and TRIF signaling pathways [23,24,25,26,27]. The MyD88-dependent pathway involves TLR4 interaction with MyD88, which then recruits tumor-necrosis-factor-receptor-associated factor 6 (TRAF6) and IL-1R-associated kinases (IRAK) family members (Figure 1). Activation of TRAF6 allows for phosphorylation of I-κB in the cytoplasm, resulting in the nuclear translocation of the transcription factor NF-ĸB into the nucleus. TRAF activation also results in the activation of MAP kinases and the production of Fos and Jun, which dimerize to form the transcription factor AP1. These transcription factors activate the transcription of genes encoding a variety of pro-inflammatory cytokines, chemokines, COX2, and iNOS [18,28,29]. The MYD88-independent (TRIF-dependent) pathway involves TLR4 interaction with TRIF, which then recruits the TRIF-related adaptor molecule (TRAM), allowing for the activation of the transcription factor interferon regulator factor 3 (IRF3). The transcription factor NF-ĸB is also activated in the TRIF-dependent pathway, but the kinetics of activation are slower than that observed in the MyD88-dependent pathway [18,28]. IRF3, the related IRF7 and NF-κB activate the transcription of genes that encode Type I Interferons such as IFN-β.

In the current study, the role of TLR4 and downstream MyD88- and TRIF-dependent signaling pathways on ethanol-induced neuroinflammation was evaluated in wild-type mice as well as mice deficient in TLR4, MyD88, or TRIF. This study further defines the molecular mechanisms by which ethanol induces neuroinflammation and neuropathology in AUD, which may result in better-targeted therapies for AUD.

## 2. Materials and Methods

### 2.1. Animals

**Figure 2:**
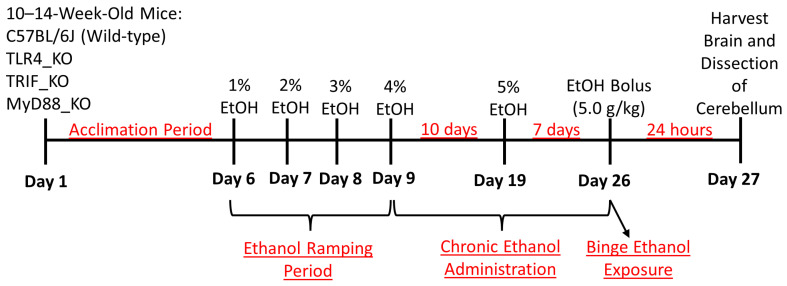
Experimental design for the adult chronic plus binge liquid diet model of AUD.

Wild-type (WT), TLR4-, MyD88-, and TRIF-global knockout (KO) mice were purchased from Jackson Laboratories (Bar Harbor, ME, USA) [30,31,32,33] (Table 1), and breeding colonies were established to produce experimental animals. All mice were bred in-house at the University of Arkansas for Medical Sciences (UAMS) in the federally approved Division of Laboratory Animal Medicine (DLAM) vivarium. All animal use protocols were approved by the UAMS Institutional Animal Care and Use Committee (IACUC). Breeders were housed on a 14:10 h light:dark cycle under controlled temperature and humidity conditions in ventilated racks, with 2 females and 1 male per cage and standard bedding, nesting material, and a polycarbonate igloo. Pups were weaned at 21 days of age and housed with 2 to 5 mice per cage until the time of experimental assignment. When male animals from each genotype reached 10 to 14 weeks in age and at least 20 g in weight, they were assigned to one of two experimental groups [Control (C) or Ethanol (E)]. At this time, experimental animals were housed individually in static cages on open-air racks, with standard bedding changed by laboratory staff weekly or as needed. Solid food was removed from the cages and water was provided *ad libitum* for the duration of the experiment. To begin the chronic plus binge ethanol treatment paradigm (Figure 2), mice were acclimated to the liquid diet on study days 1–5. The liquid diet was provided *ad libitum* via graduated glass feeding tubes using the Bio-Serv Rodent Liquid Diet, Control formulation (Flemington, NJ, USA, #F1259SP), which was prepared according to the manufacturer’s specifications. Following the 5-day acclimation period, the E group underwent a “ramping” period, in which animals received the Bio-Serv Ethanol formulation (#F1258SP) with either 1%, 2%, or 3% ethanol on study days 6, 7, and 8, respectively. The ethanol formulation was prepared using 95% *v*/*v* ethanol (ThermoFisher Scientific, Waltham, MA, USA, #AC615110010, ACS grade). On study day 9, the E-treated mice began receiving 4% ethanol for 10 days followed by 5% ethanol for an additional 7 days, which is described as the chronic ethanol administration period. Beginning on the second day of 4% ethanol administration (study day 10), the C mice were pair-fed an equivalent volume of a control diet to match the mean ethanol diet volumes consumed by the E mice on the previous day. Each afternoon around 3–5 PM CST, the liquid diets were changed. On the morning of study day 26, which is the end of the chronic administration, E mice underwent an acute “binge” administration of ethanol via gavage. At this time, the liquid diet feeding tubes were removed and standard food pellets were provided. The E-treated mice received 5 g/kg of 31.5% ethanol (*v*/*v*) diluted from 95% *v*/*v* ethanol in water, and the C group received 45% (*w*/*v*) Maltose Dextrin (Bio-Serv, 10DE Food Grade #3585) diluted in water. To ensure ethanol-treated mice recovered from the binge administration of ethanol, mice were monitored closely. Twenty-four hours following the ethanol binge administration, mice were euthanized, and tissues were harvested. This study had 17 control and 13 ethanol wild-type mice, 12 control and 12 ethanol TLR4_KO mice, 12 control and 14 ethanol MyD88_KO mice, and 10 control and 11 ethanol TRIF_KO mice. All mice used in the current study were male because our previous study indicated that ethanol induced the expression of the molecules currently studied (IL-1β, TNF-α, CCL2, and COX2) similarly in the male and female mouse cerebellum [6]. Blood ethanol concentrations were determined from a separate group of identically treated animals using an Analox AM1 alcohol analyzer as we have previously described. BECs were 230 (±59.7) mg/dL immediately following the end of the dark cycle on the last day of 4% ethanol treatment; 311.7 (±49.8) mg/dL at the end of 5% ethanol treatment; and 718 (±6.9) mg/dL 90 min following ethanol bolus [6].

### 2.2. Isolation of RNA and cDNA Synthesis

Mice were anesthetized with isoflurane vapor followed by transcardial perfusion with 1X phosphate-buffered saline (PBS) containing 5 U/mL of heparin. Once perfusion was complete, the brain was removed, and the cerebellum was microdissected and immediately flash-frozen with liquid nitrogen and stored at −80 °C. To begin RNA isolation, frozen cerebellar tissues were rapidly thawed and homogenized in Qiazol with 0.5 mm glass beads (Qiagen, Germantown, MD, USA, #13116-50) using a PowerLyzer 24 homogenizer (Qiagen #13155) for 30 s at 3500 rpm, cooled on ice, and then repeated for an additional 30 s. The miRNeasy Mini kit (Qiagen #217084) was used to isolate total RNA with DNA removed using an on-column DNase1 digestion step (Qiagen #79254), following the manufacturer’s protocol. The isolated RNA concentration was quantified using a NanoDrop 2000 spectrophotometer (Thermo Fisher Scientific, RRID:SCR_018042), and cDNA was synthesized using a Bio-Rad iScript cDNA synthesis kit (Hercules, CA, USA; Cat #1708891). According to the manufacturer’s instructions, 2 μg of RNA was diluted to 100 ng/μL in 20 μL of water and 20 μL reverse transcriptase mastermix. Samples were placed into a thermocycler and run using the suggested conditions listed in the kit. Once synthesis was completed, cDNA was diluted to 25 ng/μL and stored at 4 °C until further use.

### 2.3. Real-Time Quantitative PCR Analysis

mRNA gene expression was evaluated by quantitative real-time PCR (qRT-PCR) utilizing a Bio-Rad CFX Opus 96 Real-time PCR Detection System (Bio-Rad) and TaqMan Gene Expression Assays (Thermo Fisher Scientific #4331182) (Table 2). Duplicate 20 μL qRT-PCR reactions were run in 96-well PCR plates (Bio-Rad #HSP9601B) containing 10 μL 2X SsoAdvanced Universal Probes Supermix (Bio-Rad #1725285), 1 μL 20X TaqMan Assay, 3 μL 25 ng/μL cDNA template, and 6 μL nuclease-free water for 40 cycles (95 °C for 40 s, 60 °C for 20 s) following a hot start of 95 °C for 30 s. Mean CT values were generated for duplicate reactions and expressed as mean ∆CT relative to duplicate β-actin control reactions on the same plate for each sample. The ∆∆CT method was used to calculate relative fold differences between experimental groups when compared to control for each genotype.

### 2.4. Statistical Analysis

GraphPad Prism 9 (San Diego, CA, USA) was used to perform statistical analyses and to construct figures. Briefly, relative fold change values (∆∆CT) were organized into columns for each treatment group by genotype and were tested for normality. Likely outliers were identified using Prism’s ROUT test, Q = 1%, and excluded. Ordinary two-way analysis of variance (ANOVA) was utilized to evaluate the main effects of treatment (control or ethanol) and genotype (wild-type or TLR4_KO, MyD88_KO, or TRIF_KO) as well as the interaction between the two factors. Tukey’s multiple comparisons test was used to test for significant differences between means and *p* values were adjusted for multiple comparisons. *p* values < 0.05 were considered significant. Figures are represented as mean +/− SEM.

## 3. Results

**Figure 3:**
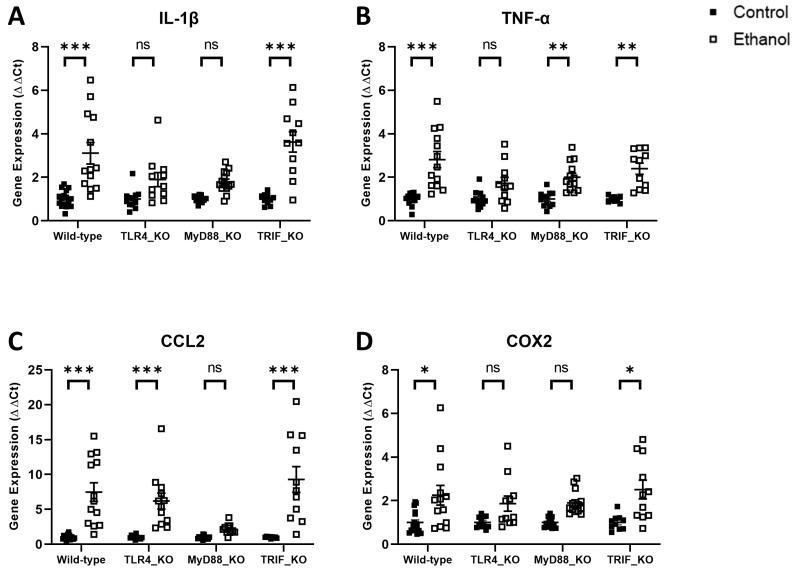
Effects of ethanol on IL-1β (**A**), TNF-α (**B**), CCL2 (**C**), and COX2 (**D**) mRNA expression in the cerebellum of wild-type, TLR4-, MyD88-, and TRIF-deficient mice. Mice were given access to control or ethanol liquid diet, as described in Methods, and relative mRNA expression was measured using qRT-PCR. Results were expressed as ∆∆CT fold change relative to control for each genotype. Individual values for each sample were plotted and error bars denote mean +/− SEM. *** *p* < 0.001, ** *p* < 0.01, * *p* < 0.05, ns = not significant.

In order to begin to define the mechanisms by which ethanol induces neuroinflammation in this adult model of AUD, we evaluated the effects of ethanol on the expression of pro-inflammatory molecules in wild-type mice versus those deficient in TLR4, MyD88, or TRIF. Two-way ANOVA was first used to evaluate the effect of ethanol treatment and genotype in wild-type versus TLR4_KO mice. Significant variance was observed for the factor of treatment for IL-1β, TNF-α, CCL2, and COX2. Genotype and interaction factors were also significant for IL-1β and TNF-α, but not for CCL2 or COX2 (Table 3). Tukey’s multiple comparisons test revealed that ethanol-treated wild-type mice had significant increases in mRNA gene expression for IL-1β (*p* < 0.001), TNF-α (*p* < 0.001), CCL2 (*p* < 0.001), and COX2 (*p* < 0.05) compared to wild-type controls (Figure 3), which is consistent with our previous study using this chronic plus binge model of AUD [6]. Ethanol-treated TLR4_KO mice, on the other hand, were not statistically different from their control group for the expression of IL-1β (*p* = 0.19), TNF-α (*p* = 0.17), or COX2 (*p* = 0.21). CCL2 expression was significantly increased (*p* < 0.001) in ethanol-treated TLR4_KO mice compared to TLR4_KO controls (Figure 3). These results suggest that TLR4 is critical in ethanol induction of neuroinflammation in our model. This is consistent with previous studies in a model of AUD in which mice were treated for 5 months with ethanol [4,5,34].

We next evaluated whether ethanol increases neuroinflammation in mice deficient in molecules critical to the TLR4 downstream signaling pathways, which are MyD88-dependent or TRIF-dependent. Two-way ANOVA was used to evaluate the effect of ethanol treatment and genotype in wild-type versus MyD88_KO mice. Significant variance was observed for the factor of treatment for IL-1β, TNF-α, CCL2, and COX2. Genotype and interaction factors were also significant for IL-1β and CCL2, but not for TNF-α or COX2 (Table 3). Tukey’s multiple comparisons test revealed that ethanol-treated MyD88_KO mice had no significant increases in mRNA gene expression for IL-1β (*p* = 0.14), CCL2 (*p* = 0.64), or COX2 (*p* = 0.06) compared to MyD88_KO controls (Figure 3). TNF-α expression was significantly increased (*p* < 0.01) in ethanol-treated MyD88_KO mice compared to MyD88_KO controls but to a lesser degree than in wild-type mice (Figure 3). Similarly, two-way ANOVA was also used to evaluate the effect of ethanol treatment and genotype in wild-type versus TRIF_KO mice; the effect of treatment was found to be significant for IL-1β, TNF-α, CCL2, and COX2. Genotype and interaction factors were not significant for any of these molecules (Table 3). Furthermore, Tukey’s multiple comparisons test revealed that ethanol-treated TRIF_KO mice had significant increases in mRNA expression for IL-1β (*p* < 0.001), TNF-α (*p* < 0.01), CCL2 (*p* < 0.001), and COX2 (*p* < 0.05) compared to TRIF_KO controls. These increases due to ethanol treatment are consistent with the increases observed in wild-type mice. Collectively, these results suggest that ethanol induces neuroinflammation through a MyD88-dependent and possibly not a TRIF-dependent signaling pathway.

**Figure 4:**
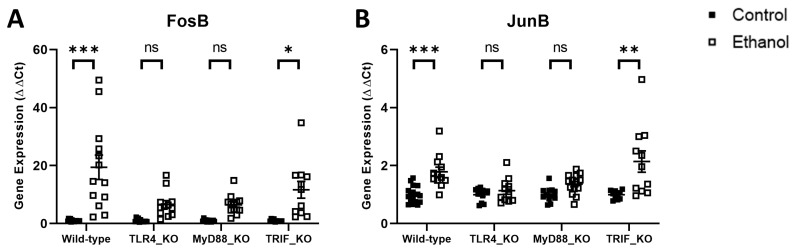
Effects of ethanol on FosB (**A**) and JunB (**B**) mRNA expression in the cerebellum of wild-type, TLR4-, MyD88-, and TRIF-deficient mice. Mice were given access to a control or ethanol liquid diet, as described in the Methods, and relative mRNA expression was measured using qRT-PCR. Results are expressed as ∆∆CT fold change relative to control for each genotype. Individual values for each sample were plotted and error bars denote mean +/− SEM. *** *p* < 0.001, ** *p* < 0.01, * *p* < 0.05, ns = not significant.

AP1 is a transcription factor that functions as a dimer of Fos and Jun proteins and is produced as an end-product of the MyD88-dependent signaling pathway. Ethanol effects on FosB and JunB were evaluated in wild-type versus TLR4-, MyD88-, or TRIF-deficient mice in a manner consistent with the pro-inflammatory molecules detailed above. Two-way ANOVA revealed significant variance for the factors of treatment and genotype as well as their interaction for FosB and JunB mRNA expression in both wild-type versus TLR4_KO mice and wild-type versus MyD88_KO mice. Only the factor of treatment was found to be significant for FosB and JunB in wild-type versus TRIF_KO mice (Table 4). Tukey’s multiple comparisons test demonstrated that ethanol significantly induced the mRNA expression of FosB and JunB in ethanol-treated wild-type mice (*p* < 0.001 and *p* < 0.001, respectively) (Figure 4). Neither FosB nor JunB were significantly different in ethanol-treated mice compared to controls in either TLR4_KO (*p* = 0.31 and *p* = 0.82, respectively) or MyD88_KO (*p* = 0.29 and *p* = 0.08, respectively) mice. Ethanol increased the expression of FosB and JunB in TRIF_KO mice compared to controls (*p* < 0.001 and *p* < 0.01, respectively), consistent with observations in wild-type mice. These results further support the role of TLR4 and downstream MyD88 signaling in ethanol-induced neuroinflammation.

**Figure 5:**
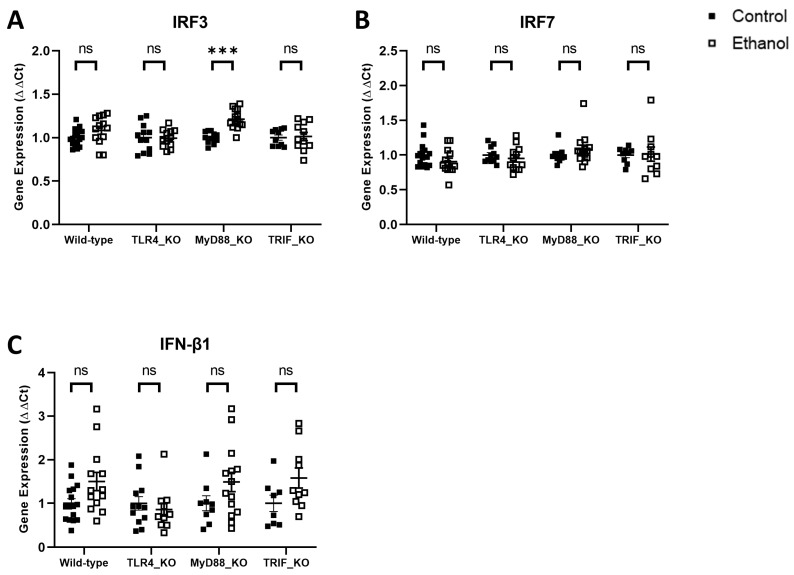
Effects of ethanol on IRF3 (**A**), IRF7 (**B**), and IFN-β1 (**C**) mRNA expression in the cerebellum of wild-type, TLR4-, MyD88-, and TRIF-deficient mice. Mice were given access to a control or ethanol liquid diet, as described in the Methods, and relative mRNA expression was measured using qRT-PCR. Results were expressed as ∆∆CT fold change relative to control for each genotype. Individual values for each sample were plotted and error bars denote mean +/− SEM. *** *p* < 0.001, ns = not significant.

The transcription factor IRF3 is an important product of the TRIF signaling pathway. IRF3 and the related transcription factor IRF7 activate the transcription of genes encoding Type I interferons, including IFN-β1. Ethanol effects on IRF3, IRF7, and IFN-β1 were evaluated in wild-type versus TLR4-, MyD88-, or TRIF-deficient mice consistent with the analyses above. Two-way ANOVA revealed no significant difference among the factors of treatment, genotype, or their interaction for IRF3 or IRF7 in wild-type versus TLR4_KO, MyD88_KO, or TRIF_KO mice, except for IRF3 in wild-type versus MyD88_KO mice (Table 5). Two-way ANOVA revealed a significant difference between the factors of genotype and interaction for IFN-β1 in wild-type versus TLR4_KO mice, and for the factor of treatment in wild-type versus MyD88_KO and TRIF_KO mice (Table 5). Tukey’s multiple comparisons test, however, demonstrated that ethanol did not significantly alter the mRNA expression of IRF3, IRF7, or IFN-β1 in ethanol-treated wild-type (*p* = 0.31, *p* = 0.42, *p* = 0.09), TLR4_KO (*p* > 0.99, *p* = 0.89, *p* = 0.93), MyD88_KO (*p* < 0.001, *p* = 0.66, *p* = 0.32), or TRIF_KO (*p* > 0.99, *p* > 0.99, *p* = 0.21) mice compared to their respective controls, with the exception of IRF3 in MyD88_KO mice (Figure 5). This further suggests that ethanol may not induce neuroinflammation through the TIRIF-dependent pathway.

## 4. Discussion

Ethanol is a commonly used addictive substance that has detrimental effects on multiple organ systems, including the CNS. AUD results in damage to multiple regions of the brain and can result in aberrant CNS function, including impaired cognition. It is appreciated that chronic ethanol abuse is associated with loss of white matter and demyelination, as well as axonal loss and neurodegeneration. Ethanol-induced neuroinflammation also occurs in AUD and is believed to contribute to neurodegeneration. Ethanol-induced neuroinflammation can also increase alcohol consumption in individuals with AUD, thus reinforcing the cycle of alcohol addiction [35,36,37].

TLR4 signaling is believed to contribute to ethanol-induced neuroinflammation. In human alcoholics, ethanol increases the expression of TLR4 and pro-inflammatory molecules such as the chemokine CCL2 [38]. Ethanol likely induces TLR4 and neuroinflammation through the production of the internal danger signal HMGB1, which acts as a ligand for TLR4 [39,40]. Animal studies further support the role of TLR4 signaling in ethanol-induced neuroinflammation. For example, TLR4 was demonstrated to play a critical role in the activation of primary cultured rodent microglia [22]. Furthermore, ethanol induced TLR4 signaling in primary rodent astrocytes through clathrin- and lipid raft-mediated receptor internalization and trafficking [41]. The role of TLR4 in ethanol induction of pro-inflammatory molecules by glia was supported by studies involving the treatment of these cells with siRNAs that block TLR4 or through the use of glia derived from TLR4-deficient mice [5,22]. Importantly, in vivo studies utilizing TLR4-deficient mice demonstrated a critical role of TLR4 in ethanol-induced neuroinflammation as well as cognitive dysfunction and anxiety behavior [34,42,43].

The current study investigated the effects of TLR4 and downstream signaling pathways in ethanol-induced neuroinflammation in an adult chronic plus binge model of AUD. We demonstrated that ethanol modulates neuroinflammation through TLR4 and the downstream MyD88-dependent, but possibly not the downstream TRIF-dependent signaling pathway. Limitations of the current study will not allow a definitive answer concerning the possible role of TRIF signaling in ethanol-induced neuroinflammation. Our results indicate that ethanol had limited effects on the expression of IRF3, IRF7, and IFNβ1, which are involved in TRIF-dependent signaling. However, additional studies involving the gain- or loss-of-function of TRIF signaling would be useful in defining a potential role of TRIF signaling in ethanol-induced neuroinflammation. In addition, the absence of ethanol-induced expression of IRF3, IRF7, and IFNβ1 alone does not prove that TRIF signaling is not involved in ethanol-induced neuroinflammation. In fact, a recent study indicates that ethanol induces IRF3/7 expression in the CNS in female rats, but not in male rats (as evaluated in male mice in the current study) [44]. An additional limitation of our study is that ethanol effects were measured at the level of mRNA, and future studies are needed to determine if ethanol alters the expression of the associated proteins. Mild chronic stress has been demonstrated to increase the expression of TLR4 and MyD88 [45,46]. Thus, since our mice are of necessity housed individually in the current study, which could produce chronic stress, future studies are needed to tease out the roles of ethanol and stress on ethanol-induced neuroinflammation. We also cannot determine from the current study if ethanol induces neuroinflammation directly on the CNS or indirectly through inducing inflammation in peripheral tissues such as liver or through effects on immune cells such as macrophages. Future studies involving knocking out TLR4 and downstream signaling molecules in microglia and astrocytes specifically would be helpful in addressing these questions. Studies designed to determine additional mechanisms through which ethanol induces neuroinflammation, such as by altering glial phenotype or altering alcohol consumption, would also be informative.

The current study suggests that TLR4 and MyD88 signaling pathways could represent important targets for the treatment of AUD. The feasibility of this therapeutic approach is supported by a variety of studies. For example, TLR agonists and antagonists have been developed that could be beneficial in the treatment of AUD [47]. In addition, agents effective in blocking HMGB1/TLR4/NF-κB signaling have proven protective in animal models of Parkinson’s disease and could be useful in the treatment of AUD [48]. Nanoparticles have been used to facilitate the movement of molecules across the blood–brain barrier, and nanoparticle formulations of TLR4shRNA-expressing plasmids have proven effective in suppressing post-ischemia inflammation as well as protection against ischemia-associated loss of sensory and motor functions [49], suggesting that they may also be effective in the treatment of AUD. MicroRNAs are capable of modulating inflammation and may be another mechanism to regulate TLR4 and downstream signaling pathways [50]. The development of potential AUD therapies that target the MyD88 signaling pathway but leave the TRIF-dependent signaling pathway unaffected could be particularly valuable. In this regard, TLR4-biased small-molecule modulators are being developed. These biased modulators may be capable of selectively activating MYD88- but not TRIF-dependent signaling, potentially leading to increased efficacy and decreased adverse side effects [51]. In addition, small-molecule inhibitors have been developed that block MyD88-dependent signaling by blocking the formation of MyD88 homodimers required for downstream signaling [52]. Macromolecular complexes play a critical role in the activation of TLR4 downstream signaling pathways. For example, Myddosomes are macromolecular complexes critical in MyD88-dependent signaling. Thus, agents that block the formation of macromolecular complexes such as Myddosomes may be effective in the treatment of AUD [53]. It is hoped that targeting TLR4 and MyD88-dependent signaling may be effective in the treatment of AUD.

## Figures and Tables

**Table 1:** Mouse strains. List of wild-type, TLR4-, MyD88-, and TRIF-deficient mice used to perform this study, along with their common name, JAX Lab strain reference number, and RRID.

Generic Name	Strain	Common Name	JAX Lab Strain	RRID
Wild-type	C57BL/6J	B6	000664	IMSR_JAX:000664
TLR4_KO	B6.B10ScN-Tlr4^lps-del^/JthJ	Tlr4^Lps-del^	007227	IMSR_JAX:007227
MyD88_KO	B6.129P2(SJL)-Myd88^tm1.1Defr^/J	Myd88 null	009088	IMSR_JAX:009088
TRIF_KO	C57BL/6J-Ticam1^Lps2^/J	Trif^Lps2^	005037	IMSR_JAX:005037

**Table 2:** TaqMan gene expression assays. 20X primer/probe sets (FAM-MGB) were purchased from Thermo Fisher Scientific, Cat. #4331182, and were used at a final concentration of 1X for qRT-PCR. Assays were selected to span an exon–exon junction where possible.

Gene Name	Assay ID
*IL-1β*	Mm00434228_m1
*TNF-α*	Mm00443258_m1
*CCL2*	Mm00441242_m1
*Ptgs2 (COX2)*	Mm00478374_m1
*Fosb*	Mm00500403_m1
*Junb*	Mm04243546_s1
*IRF3*	Mm00516784_m1
*IRF7*	Mm00516793_g1
*IFN-β1*	Mm00439552_s1

**Table 3:** Two-way ANOVA for IL-1β, TNF-α, CCL2, and COX2 in the cerebellum of wild-type, TLR4-, MyD88-, and TRIF-deficient mice. GraphPad Prism 9 was utilized to perform ordinary two-way analysis of variance (ANOVA) as described in the Methods. The main effects of treatment (control or ethanol) and genotype (wild-type or TLR4_KO, MyD88_KO, or TRIF_KO) and their interaction were evaluated. *p* values <0.05 were considered significant. *** *p* < 0.001, * *p* < 0.05, ns = not significant.

**IL-1β**	**Wild-Type vs** **. TLR4_KO**			**Wild-Type vs. MyD88_KO**			**Wild-Type vs. TRIF_KO**		
**Two-Way ANOVA**	**F (DFn, DFd)**	***p* Value**	**Sig.**	**F (DFn, DFd)**	***p* Value**	**Sig.**	**F (DFn, DFd)**	***p* Value**	**Sig.**
Interaction	F (1, 49) = 4.296	0.04	*	F (1, 52) = 7.100	0.01	*	F (1, 47) = 0.5886	0.45	ns
Genotype	F (1, 49) = 4.296	0.04	*	F (1, 52) = 7.136	0.01	*	F (1, 47) = 0.5840	0.45	ns
Treatment	F (1, 49) = 26.26	<0.001	***	F (1, 52) = 34.08	<0.001	***	F (1, 47) = 49.17	<0.001	***
**TNF-** **α**	**Wild-type vs. TLR4_KO**			**Wild-type vs. MyD88_KO**			**Wild-type vs. TRIF_KO**		
**Two-way ANOVA**	**F (DFn, DFd)**	***p* value**	**Sig.**	**F (DFn, DFd)**	***p* value**	**Sig.**	**F (DFn, DFd)**	***p* value**	**Sig.**
Interaction	F (1, 48) = 5.682	0.02	*	F (1, 50) = 3.729	0.06	ns	F (1, 46) = 0.8172	0.37	ns
Genotype	F (1, 48) = 5.635	0.02	*	F (1, 50) = 3.717	0.06	ns	F (1, 46) = 0.8123	0.37	ns
Treatment	F (1, 48) = 30.51	<0.001	***	F (1, 50) = 45.36	<0.001	***	F (1, 46) = 47.64	<0.001	***
**CCL2**	**Wild-type vs. TLR4_KO**			**Wild-type vs. MyD88_KO**			**Wild-type vs. TRIF_KO**		
**Two-way ANOVA**	**F (DFn, DFd)**	***p* value**	**Sig.**	**F (DFn, DFd)**	***p* value**	**Sig.**	**F (DFn, DFd)**	***p* value**	**Sig.**
Interaction	F (1, 49) = 0.5700	0.45	ns	F (1, 50) = 16.21	<0.001	***	F (1, 46) = 0.6718	0.42	ns
Genotype	F (1, 49) = 0.5684	0.45	ns	F (1, 50) = 16.23	<0.001	***	F (1, 46) = 0.6718	0.42	ns
Treatment	F (1, 49) = 47.53	<0.001	***	F (1, 50) = 33.55	<0.001	***	F (1, 46) = 44.86	<0.001	***
**COX2**	**Wild-type vs. TLR4_KO**			**Wild-type vs. MyD88_KO**			**Wild-type vs. TRIF_KO**		
**Two-way ANOVA**	**F (DFn, DFd)**	***p* value**	**Sig.**	**F (DFn, DFd)**	***p* value**	**Sig.**	**F (DFn, DFd)**	***p* value**	**Sig.**
Interaction	F (1, 48) = 0.4550	0.5	ns	F (1, 52) = 0.5579	0.46	ns	F (1, 47) = 0.1670	0.68	ns
Genotype	F (1, 48) = 0.4508	0.51	ns	F (1, 52) = 0.5526	0.46	ns	F (1, 47) = 0.1670	0.68	ns
Treatment	F (1, 48) = 13.56	<0.001	***	F (1, 52) = 20.60	<0.001	***	F (1, 47) = 19.05	<0.001	***

**Table 4:** Two-way ANOVA for FosB and JunB in the cerebellum of wild-type, TLR4-, MyD88-, and TRIF-deficient mice. GraphPad Prism 9 was utilized to perform ordinary two-way analysis of variance (ANOVA) as described in the Methods. The main effects of treatment (control or ethanol) and genotype (wild-type or TLR4_KO, MyD88_KO, or TRIF_KO) and their interaction were evaluated. *p* values < 0.05 were considered significant. *** *p* < 0.001, ** *p* < 0.01, * *p* < 0.05, ns = not significant.

**FosB**	**Wild-Type vs. TLR4_KO**			**Wild-Type vs. MyD88_KO**			**Wild-Type vs. TRIF_KO**		
**Two-Way ANOVA**	**F (DFn, DFd)**	***p* Value**	**Sig.**	**F (DFn, DFd)**	***p* Value**	**Sig.**	**F (DFn, DFd)**	***p* Value**	**Sig.**
Interaction	F (1, 48) = 8.583	0.005	**	F (1, 49) = 9.414	0.004	**	F (1, 45) = 2.203	0.14	ns
Genotype	F (1, 48) = 8.584	0.005	**	F (1, 49) = 9.421	0.003	**	F (1, 45) = 2.204	0.14	ns
Treatment	F (1, 48) = 30.17	<0.001	***	F (1, 49) = 32.22	<0.001	***	F (1, 45) = 31.07	<0.001	***
**JunB**	**Wild-type vs. TLR4_KO**			**Wild-type vs. MyD88_KO**			**Wild-type vs. TRIF_KO**		
**Two-way ANOVA**	**F (DFn, DFd)**	***p* value**	**Sig.**	**F (DFn, DFd)**	***p* value**	**Sig.**	**F (DFn, DFd)**	***p* value**	**Sig.**
Interaction	F (1, 48) = 8.811	0.005	**	F (1, 51) = 4.386	0.04	*	F (1, 46) = 0.8740	0.35	ns
Genotype	F (1, 48) = 8.824	0.005	**	F (1, 51) = 4.327	0.04	*	F (1, 46) = 0.8699	0.36	ns
Treatment	F (1, 48) = 18.09	<0.001	***	F (1, 51) = 32.12	<0.001	***	F (1, 46) = 25.82	<0.001	***

**Table 5:** Two-way ANOVA for IRF3, IRF7, and IFN-β1 in the cerebellum of wild-type, TLR4-, MyD88-, and TRIF-deficient Mice. GraphPad Prism 9 was utilized to perform ordinary two-way analysis of variance (ANOVA) as described in the Methods. The main effects of treatment (control or ethanol) and genotype (wild-type or TLR4_KO, MyD88_KO, or TRIF_KO) and their interaction were evaluated. *p* values < 0.05 were considered significant. *** *p* < 0.001, ** *p* < 0.01, * *p* < 0.05, ns = not significant.

**IRF3**	**Wild-Type vs. TLR4_KO**			**Wild-Type vs. MyD88_KO**			**Wild-Type vs. TRIF_KO**		
**Two-Way ANOVA**	**F (DFn, DFd)**	***p* Value**	**Sig.**	**F (DFn, DFd)**	***p* Value**	**Sig.**	**F (DFn, DFd)**	***p* Value**	**Sig.**
Interaction	F (1, 50) = 1.578	0.21	ns	F (1, 52) = 4.392	0.04	*	F (1, 47) = 0.9352	0.34	ns
Genotype	F (1, 50) = 1.578	0.21	ns	F (1, 52) = 4.279	0.04	*	F (1, 47) = 0.8840	0.35	ns
Treatment	F (1, 50) = 1.197	0.28	ns	F (1, 52) = 23.61	<0.001	***	F (1, 47) = 1.707	0.20	ns
**IRF7**	**Wild-type vs. TLR4_KO**			**Wild-type vs. MyD88_KO**			**Wild-type vs. TRIF_KO**		
**Two-way ANOVA**	**F (DFn, DFd)**	***p* value**	**Sig.**	**F (DFn, DFd)**	***p* value**	**Sig.**	**F (DFn, DFd)**	***p* value**	**Sig.**
Interaction	F (1, 48) = 0.2452	0.62	ns	F (1, 49) = 3.275	0.08	ns	F (1, 44) = 0.8061	0.37	ns
Genotype	F (1, 48) = 0.2392	0.63	ns	F (1, 49) = 3.321	0.07	ns	F (1, 44) = 0.7919	0.38	ns
Treatment	F (1, 48) = 2.433	0.13	ns	F (1, 49) = 0.0097	0.92	ns	F (1, 44) = 0.4236	0.52	ns
**IFN-** **β1**	**Wild-type vs. TLR4_KO**			**Wild-type vs. MyD88_KO**			**Wild-type vs. TRIF_KO**		
**Two-way ANOVA**	**F (DFn, DFd)**	***p* value**	**Sig.**	**F (DFn, DFd)**	***p* value**	**Sig.**	**F (DFn, DFd)**	***p* value**	**Sig.**
Interaction	F (1, 48) = 4.196	0.05	*	F (1, 48) = 0.0011	0.97	ns	F (1, 43) = 0.0469	0.83	ns
Genotype	F (1, 48) = 4.234	0.05	*	F (1, 48) = 0.0009	0.98	ns	F (1, 43) = 0.0455	0.83	ns
Treatment	F (1, 48) = 1.312	0.26	ns	F (1, 48) = 7.038	0.01	*	F (1, 43) = 8.436	0.006	**

## Data Availability

The data presented in this study are available within the article.
